# The acute diabetic foot: different clinical patterns with a dedicated fast-track pathway

**DOI:** 10.1007/s00592-026-02746-w

**Published:** 2026-07-14

**Authors:** Laura Giurato, Roberto Da Ros, Alessia Scatena, Roberto De Giglio, Giuseppe Murdolo, Marco Mastinu, Maurizio Gicchino, Desiree Gabriele, Cesare Miranda, Luigi Uccioli, Marco Meloni

**Affiliations:** 1 Division of Endocrinology and Diabetes, Department of Surgical Sciences, CTO Hospital, University of Rome Tor Vergata, Rome, Italy; 2 Diabetes and Diabetic Foot Treatment Center, ASUGI, Monfalcone, Italy; 3 Diabetology Unit, Health Authorities South East Tuscany, San Donato Hospital, Arezzo, Italy; 4 Diabetic Foot Functional Unit, San Carlo, Paderno Dugnano, Milan, Italy; 5 Department of Endocrinology and Metabolism, Santa Maria Della Misericordia Hospital, Perugia, Italy; 6 Unit of Metabolic Diseases and Diabetology, San Martino Hospital, Oristano, Italy; 7 Unit of Endocrinology and Metabolic Diseases, University Hospital L. Vanvitelli, Naples, Italy; 8 University of Rome Tor Vergata, Rome, Italy; 9 Clinic of Endocrinology and Metabolism Diseases, ASFO, Pordenone, Italy; 10 Diabetic Foot Centre, Unit of Endocrinology and Diabetology, Department of Systems Medicine, University of Rome Tor Vergata, Rome, 00133 Italy

**Keywords:** Diabetic foot, Diabetic foot ulcers, Diabetic foot attack, Limb salvage, Fast-track pathway

## Abstract

Although scientific societies have made substantial efforts through the implementation of guidelines and by increasing awareness among both expert and non-expert healthcare professionals (HCPs), delayed referral of patients with diabetic foot ulcers (DFUs) to specialized diabetic foot services (DFS) remains a common issue. Early referral to a DFS has been widely documented as the most important intervention for achieving ulcer healing, reducing healing time, and avoiding major amputations. Furthermore, it has been reported that many HCPs working in primary care are not adequately trained in the recognition and management of DFUs. The onset of a DFU may rapidly deteriorate and evolve into a diabetic foot attack (DFA), a dangerous condition characterized by advanced ischaemia, severe infection, and/or acute Charcot neuroarthropathy, potentially leading to limb- and life-threatening consequences. The current document aims to identify the main clinical patterns of DFA, describing their clinical characteristics, appropriate management pathways, and indications for treatment. Accordingly, the authors aimed to develop a practical, fast-track model to define referral timing to specialized diabetic foot centers based on the severity of each clinical presentation. The proposed fast-track approach distinguishes between emergency conditions requiring immediate management within hours and urgent conditions requiring specialist evaluation within 24–48 h to prevent clinical deterioration and poorer outcomes.This document was developed on behalf of the Italian Diabetic Foot Study Group of Società Italiana di Diabetologia (SID) and Associazione Medici Diabetologi (AMD) with the aim of reducing cases of delayed referral and their severe consequences.

**Supplementary Information:**

The online version contains supplementary material available at https://doi.org/10.1007/s00592-026-02746-w.

## Introduction

Diabetic foot disease (DFD) is considered a devastating complication of diabetes and a recognized risk factor for amputation, mortality, and hospitalization, with a significant impact on the healthcare burden [1].

The lifetime risk of developing a diabetic foot ulcer (DFU) in persons with diabetes is between 19% and 34% [2], thereforte it should be considered a common long-term complication of diabetes. DFUs have different patterns, and it is mandatory for healthcare professionals (HCPs) to identify acute conditions and the most serious frameworks that usually require urgent treatment and often necessitate hospitalization.

The ‘diabetic foot attack’ (DFA) is the most severe presentation of DFD, which is typically characterized by the onset of an acutely inflamed foot with a progressive evolution that threatens both limb and life [3]. The DFA can be distinguished in three forms: ischemic foot, in the case of acute limb ischaemia (ALI) or quick impairment of chronic limb-threatening ischaemia (CLTI); infected foot that may be often a complication of chronic wound, which can occur with or without a concomitant ischaemia; acute Charcot Neuroarthropathy, defined by a rapid deterioration secondary to a bone collapse and often complicated by infected ulcers [3, 4].

Within the fast-track framework, acute diabetic foot attack (DFA) is primarily defined by ischaemia, infection, and acute Charcot neuroarthropathy. A foot ulcer, as an acute event, may be considered within the spectrum of diabetic foot attack (DFA), as it often represents the initial clinical substrate through which infection or tissue necrosis develops. If not promptly recognized and appropriately managed, an apparently simple ulcer can rapidly evolve into a limb-threatening condition, reflecting the time-dependent nature of the disease process.

Although a huge work of scientific communities through the implementation of guidelines and awareness among expert and less expert HCPs, delayed referral of patients affected by DFUs to a specialised diabetic foot team (DFT) is still a common issue [5]. In more than 60% of cases, the duration of DFU is unknown, or its diagnosis is delayed more than three weeks from the onset of the wound [5]. Over 50% of DFUs are complicated by infection or ischaemia at presentation, substantially increasing the risk of amputation, hospitalization, and mortality [5, 6]. Early referral to specialised DFT has been widely documented as the most important intervention for achieving healing, reducing healing time, and decreasing major amputations [7].

It has also been reported that up to 50% of cases, HCPs working in primary care or general practitioners (GPs) are not adequately trained in the recognition and basic management of DFD [8].

Recently, a survey conducted by the Italian diabetic foot study group involving approximately one hundred diabetes services addressed to diabetic foot care, reported that delayed referral to specialised DFT and urgent surgery were the main barriers in the management of patients affected by DFUs [9].

## Objectives and methods

The present document aims to identify the most important patterns of DFA, describing their clinical characteristics, the appropriate management pathway, and indications for treatment, specifically during the acute phase, including timing of referral to specialized DF centers.

As reported above, acute diabetic foot encompasses ischaemia, infection, and acute Charcot neuroarthropathy.

In their advanced stages, these clinical conditions define the DFA. They are time-dependent, and any delay in recognition and management markedly increases the risk of major complications and limb loss [3].

Accordingly, the authors aimed to develop a model to define referral timing to specialized diabetic foot centers, based on the severity of each clinical presentation.

This fast-track approach distinguishes between emergency conditions requiring immediate management within hours and urgent conditions requiring specialist evaluation within 24–48 hours to prevent clinical deterioration and limb-threatening complications. We also identified diabetic foot ulceration as the initiating event leading to more severe DFA frameworks, as its progression and complications most commonly represent the onset of any DFA pattern.

The document has been developed on behalf of the Italian Diabetic Foot Study Group of SID (Società Italiana Diabetologia) and AMD (Associazione Medici Diabetologi) to provide a practical tool for healthcare professionals working in community and primary care settings, with the aim of reducing delayed referrals and their severe consequences, such as hospitalization, amputation, and impaired quality of life.

### Diabetic foot ulcer

The onset of foot ulcers in persons with diabetes is very common, affecting up tp 34% of subjects with this specific metabolic disease [2]. DFU is usually a combination of peripheral neuropathy (loss of peripheral sensitivity), peripheral arterial disease (PAD), and foot deformities. The onset of a foot ulcer in diabetes should always be considered a deadly condition that requires early evaluation, identification of potential conditions that can quickly impair the local damage, and starting treatment before referring patients to a specialized DFS.

Different wound characteristics can influence the outcomes of patients with DFUs, something related specifically to the healing process, something else related to the risk of minor/major amputation.

**Fig. 1:**
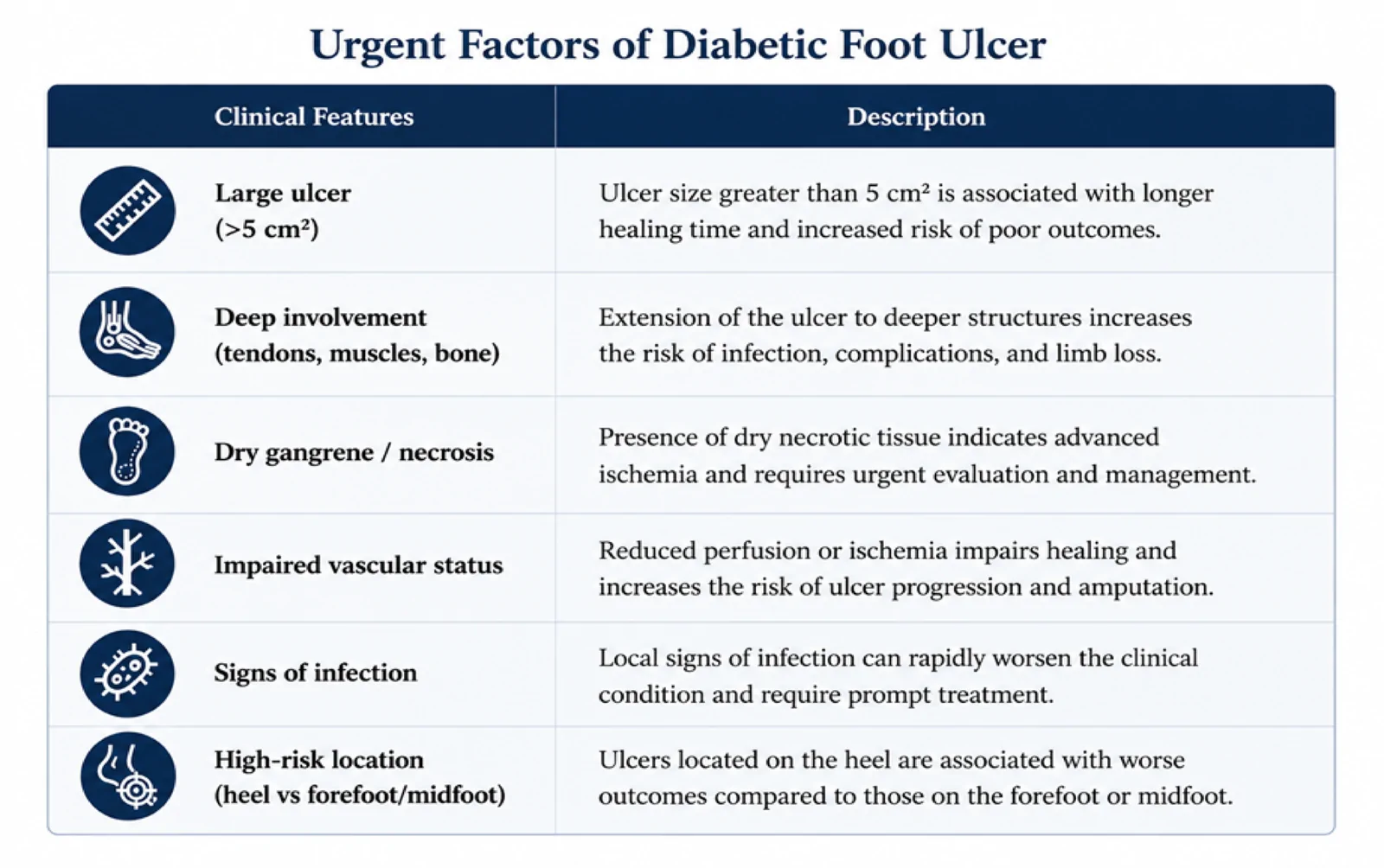
Urgency factors to be considered in persons with the onset of diabetic foot ulcer and related management

The wound features that need a detailed evaluation are usually size, depth, presence of gangrene, vascular status, infection, and location (Fig. 1).

Size (ulcer > 5 cm^2^) and depth (ulcer involving tendon, muscle, and/or bones) are two elements related to non-healing or longer healing time [10]. The presence of a deep wound involving tendons, muscles, and bone could often require surgical intervention or advanced technologies that are usually available only in specialised settings. The presence of gangrene is usually related to a local ischaemia coming from a poor blood perfusion of an infectious process determining an ischaemic condition. The presence of gangrene requires an expert and dedicated evaluation of a specialised DFS both for defining the need for vascular treatment and for surgical treatment (minor amputation or removal of an extensive part of the foot) [11].

Vascular status should be evaluated by the clinical history (cardiovascular risk factors, previous vascular interventions), the presence of pulses, and easy bed tests when available (ankle-brachial index, toe-brachial index, toe pressure, ankle pressure, wave doppler) [12]. The suspicion of impaired vascular perfusion needs early evaluation and treatment. Ischaemia is one of the main risk factors for non-healing, longer healing time, amputation, and also mortality [10, 13, 14]. The presence of infection should be clinically evaluated [15].

Finally, the location of the foot ulcer represents a key indicator of local severity. The majority of DFUs (nearly to 90%) are located in the forefoot [10, 16], but the heel location is related to worse outcomes such as non-healing, long healing time, and higher risk of major amputation [11, 16–20]. In addition, heel ulcers are often associated with poor vascular status and underlying bone involvement, and these conditions usually require a complex multi-disciplinary and surgical approach that includes vascular intervention, demolitive and reconstructive surgery. Therefore, patients affected by heel DFUs require careful observation and usually a specialised DFT for their management.

Even though critical episodes or clinical frameworks that could impact on outocomes need specialised DFS for deeper investigations and multi-disciplinary management, every condition requires a basic and starting treatment at first assessment. Any DFUs should be treated in line with documented guidelines which need at least the empirical management of infection, offloading for reducing ulcer pressures, adequate wound care and monitoring of diabetes, diabetic complications and comorbidities [21].

### Ischaemic diabetic foot

The last two decades have documented significant changes in diabetic foot disease (DFD), with an increase in ischemic DFUs compared with neuropathic DFUs, reaching up to 70% in some settings [13, 22]. Ischaemic foot disease is associated with worse outcomes, with an increased risk of non-healing and major amputation [10, 13, 14], especially in the presence of concomitant infection [10].

Ischaemic DFUs are usually characterized by necrosis, gangrene, or chronic wounds that fail to show signs of healing, in the absence of other limiting factors (e.g., infection, lack of offloading, or inadequate wound care). Typically, ischemic wounds are characterized by absent foot pulses; however, their presence does not completely exclude PAD, particularly in the presence of the above findings or a suggestive clinical history (long diabetes duration, ischemic heart disease, end-stage renal disease, smoking, or previous vascular intervention) [12, 21].

Rest pain may be present in some cases but is often absent in patients with concomitant peripheral neuropathy and reduced sensitivity. Bedside tests may be useful to confirm clinical suspicion (ankle–brachial index, toe–brachial index, toe pressure, ankle pressure, Doppler waveforms). In addition, color duplex ultrasound can better define the presence, location, and extent of stenoses and occlusions in the peripheral arterial tree [12]. The main patterns that define a DFA are the acute limb ischaemia (ALI) and the progression of chronic limb-threatening ischaemia (CLTI) [3, 4] (Table 1).

**Table 1:** Ischaemic foot

Acute limb ischaemia (ALI)
• Acute presentation of necrosis/gangrene • Blisters • Hyperemia or cyanosis around the wound • Resting pain
**Progression of Chronic Limb Threatening Ischaemia (CLTI)**
• Progression/extension of chronic wound (necrosis, dry gangrene, stable wound) • Hyperemia or cyanosis around the wound (less frequent than ALI) • Resting pain (unusual)

ALI is a less frequent condition, usually related to thrombotic events in large vessels, mainly involving infra-popliteal or below-the-ankle arteries, or, in some cases, embolic events leading to acute occlusion of distal digital arteries. This condition carries a high risk of gangrene, which may progressively involve the toes, forefoot, and, in the most severe cases, the entire foot [23–25].

**Fig. 2:**
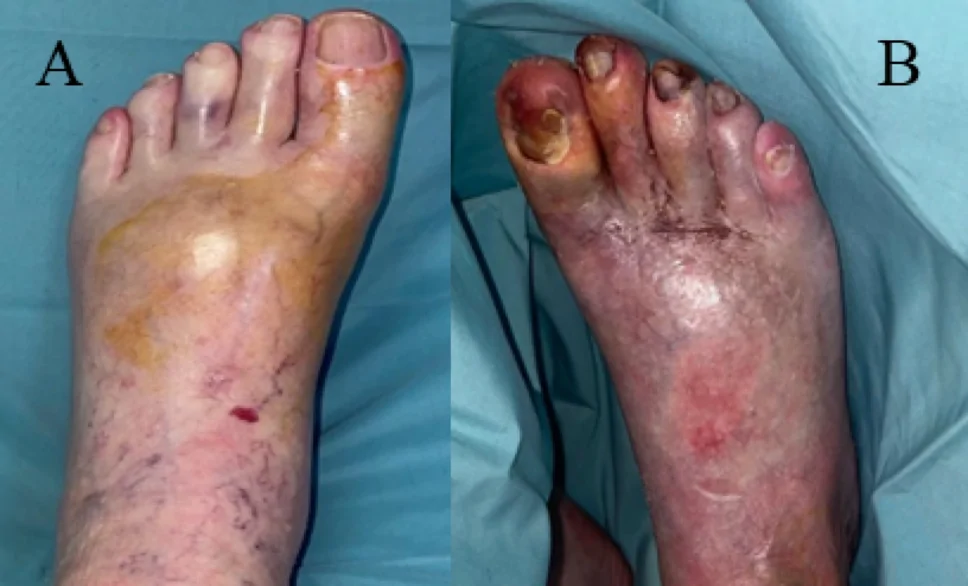
**A** Acute limb ischaemia in a patient with sudden onset of digital lesions. **B** Acute limb ischaemia in a patient without foot lesions

The clinical presentation of ALI may vary, and initial skin changes may include blisters or vesicles, in addition to necrotic wounds, gangrene, or ischemic ulcers without necrotic tissue. The foot may present with hyperemia, cyanosis, pallor around the wound(s), or areas of necrosis, all of which are signs of acute ischemic injury [26] (Fig. 2a, b). Pain is more frequently present than in CLTI.

**Fig. 3:**
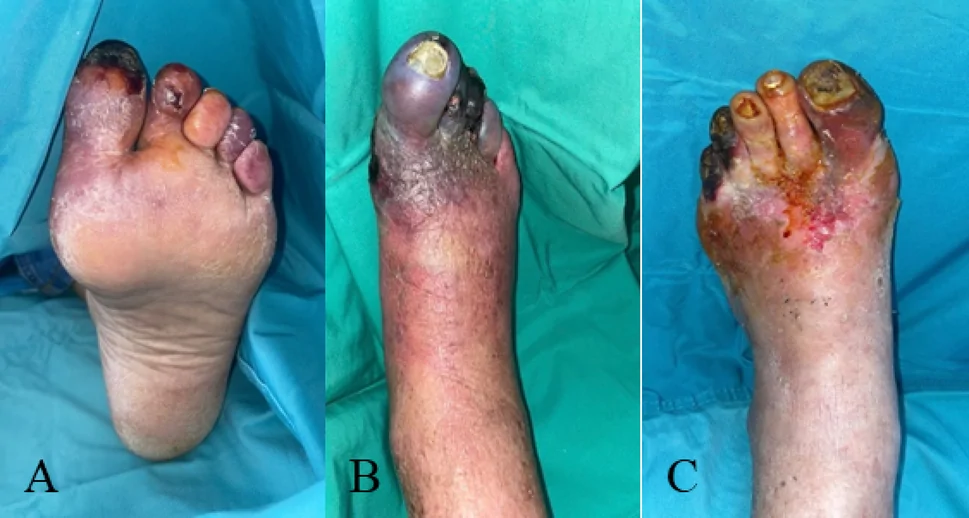
**A**,** B**,** C.** Quick progression ischaemic framework in patients with a history of chronic limb ischaemia

The progression of CLTI is usually characterized by a rapid enlargement in terms of size and depth of ischemic lesions, necrotic wounds, and/or gangrene involving large areas of the foot, leading to an increased risk of minor amputation or extensive ablative surgery (Fig. 3a–c). This second pattern of “ischemic foot attack” may also present as a stable wound associated with increased pain (usually rest pain), requiring early management due to impaired quality of life.

ALI, and more commonly CLTI, may be associated with concomitant infection, which represents an additional factor contributing to ischemic deterioration and, in these cases, both of them require more urgent management [15, 27]. Infection can be recognized by the presence of wet gangrene or specific inflammatory signs (see the infection section for further details). In cases of ALI or clinical progression of CLTI, patients should be promptly referred to a specialized DFS for urgent assessment and lower-limb revascularization [7]. Early revascularization in these cases is essential for limb salvage, reduction of major amputation, and improved healing outcomes [12, 27, 28].

Urgent referral and hospitalization in a dedicated DFS are also required in cases of concomitant infection, which is associated with a higher risk of adverse outcomes. In these cases, broad-spectrum antibiotic therapy should be initiated immediately, even during the transfer to a specialized center [7].

### Infected diabetic foot

Infection in the diabetic foot develops in the presence of a pre-existing ulcer, resulting from bacterial proliferation within the affected tissues and leading to a clinically evident inflammatory response.

Infection complicates DFU in 50–60% of cases; approximately one in five moderate to severe infections requires hospitalization and can lead to lower limb amputation [29–31].

Infection is diagnosed by the presence of at least two of the following local signs: erythema, warmth, swelling or induration, pain or tenderness, and purulent discharge [32]. Osteomyelitis is a frequent and clinically relevant complication of soft tissue infection [33].

The depth and anatomical extent of infection are major prognostic factors, distinguishing superficial from deep infection as well as localized from systemic disease [34, 35].

Optimal outcomes rely on early identification, accurate diagnosis, and timely management, including appropriate microbiological sampling, empiric antimicrobial therapy followed by targeted treatment, and prompt recognition of cases requiring surgical source control. Delays in recognizing severe or progressive infection remain a key modifiable factor associated with poor outcomes [36].

Infection is a key driver of clinical deterioration, frequently originating from a pre-existing ulcer and extending to deeper structures, including soft tissues and bone. Characteristic features include rapidly spreading inflammation, tissue necrosis, abscess formation, and the possible presence of soft tissue gas, with or without systemic signs [3, 37]. Life-threatening conditions are those that pose an immediate risk to the patient’s survival (e.g., sepsis or septic shock), while limb-threatening conditions are severe limb complications that may lead to amputation if not promptly treated (e.g., critical ischaemia or deep infection) [34]. 

Early recognition of DFA and prompt referral to a specialized DFC are essential for rapid infection control, timely surgical management, and coordinated care, reducing the risks of irreversible tissue damage, major amputation, and sepsis-related death [38, 39].

The most severe presentations include abscess or phlegmon, gangrene, necrotizing fasciitis and compartment syndrome [40–43].

The most moderate-severe infectious presentations (without systemic involvement) **requiring urgent referral t**o a diabetic foot center include abscess, phlegmon, gangrene, and acute osteomyelitis (Table 2).

**Table 2:**
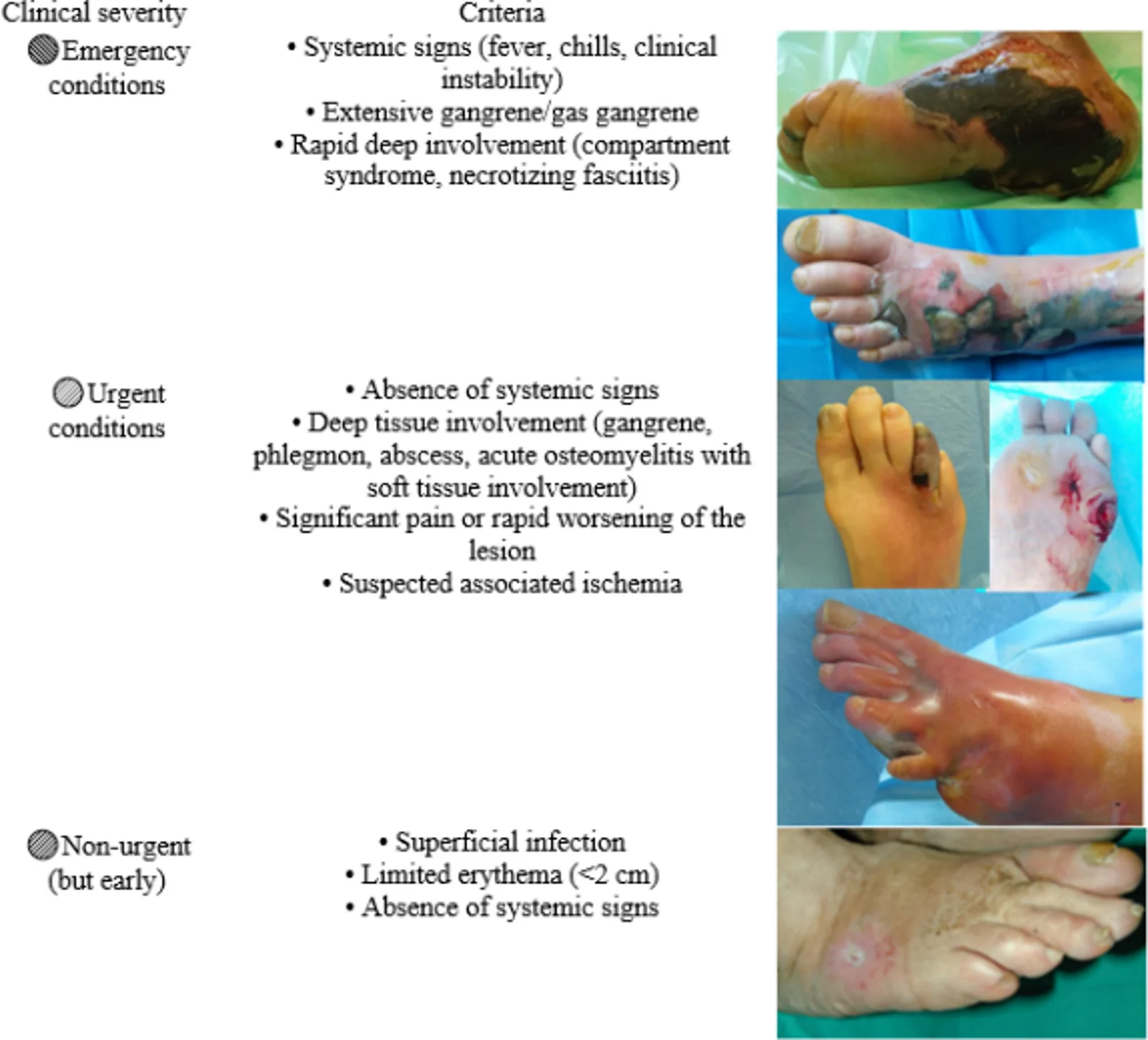
Clinical manifestations and criteria for infection severity

**Abscess** is a localized collection of purulent material, typically confined to the dermis and subcutaneous tissues. Clinical findings suggestive of its presence include purulent drainage from an ulcer, a well-demarcated surrounding area of cellulitis, and fluctuation of the underlying soft tissues. Although localized, an abscess represents a high-risk condition due to its potential for rapid extension if not promptly treated.

**Phlegmon** represents a diffuse, non-encapsulated spreading infection of the soft tissues, involving a broader tissue plane than a localized abscess. It is characterized by poorly defined inflammatory infiltration and extension along fascial planes, often without clear anatomical boundaries. This pattern reflects an aggressive infectious process requiring urgent evaluation and management.

**Gangrene** is defined by tissue necrosis secondary to critical infection and/or ischaemia. It is characterized by skin discoloration ranging from dusky purple to black, soft tissue swelling, and variable presence of purulent collections depending on whether the process is dry or wet gangrene. It represents a limb-threatening condition requiring urgent surgical and medical management.

**Acute Osteomyelitis with associated soft tissue involvement** represents a severe deep infection in which bone infection is accompanied by contiguous spread to the surrounding soft tissues, often through chronic ulcers. It is characterized by persistent inflammation and may present with exposed bone, sinus tract formation, and surrounding cellulitis, requiring combined medical and surgical management.

The most severe infectious presentations associated with systemic involvement **require emergency referral** to a diabetic foot center and include gas gangrene, necrotizing fasciitis, and compartment syndrome (Table 2).

**Gas gangrene** (clostridial myonecrosis) is a rapidly progressive, life-threatening soft tissue infection with gas formation and muscle necrosis. It presents with severe pain (not always present in case of neuropathy), swelling, and crepitus, and requires immediate surgical debridement and antibiotics.

**Necrotizing fasciitis** is a rapidly progressive deep soft tissue infection involving the fascial planes and subcutaneous tissues. It typically presents with severe pain, erythema, edema, and rapidly evolving skin discoloration, progressing to extensive necrosis of skin, subcutaneous tissue, and fascia. It is frequently associated with systemic toxicity and high mortality if not promptly treated.

**Compartment syndrome** is a limb-threatening condition caused by increased pressure within a closed fascial compartment, leading to impaired perfusion and rapid tissue ischaemia. It presents with severe, disproportionate pain, tense swelling, and evolving neurological deficits, and requires immediate surgical decompression to prevent irreversible damage and limb loss [3, 30].

Severe bone infections are frequently associated with diabetic foot attack, worsening its clinical course and requiring aggressive management, including surgical debridement and prolonged antibiotic therapy, with possible progression to limb amputation [27, 44].

In DFA, severe infection or moderate infection complicated by critical features such as extensive gangrene, necrotizing infection, deep (subfascial) abscess, compartment syndrome, requires urgent surgical assessment and prompt referral to a specialized diabetic foot center. These conditions should be considered surgical emergencies, as they are associated with rapid clinical deterioration and a high risk of tissue loss, and often require early surgical intervention within 24–48 h in combination with antibiotic therapy to ensure adequate source control [30].

### Acute charcot foot

Acute Charcot foot represents one of the most critical and, unfortunately, still underestimated clinical challenges within the context of diabetes complications and peripheral neuropathies. 

Variable Prevalence: In the general diabetic population, prevalence is low, estimated between 0.1% and 0.4%. The annual incidence is estimated at 1.2 to 8.5 cases per 1,000 people with diabetes [45].

Primary Risk Factors:

- Neuropathy: present in 100% of subjects, it is the fundamental prerequisite.
- Duration of diabetes: typically manifests after at least 10–20 years of diabetes history [46].
- Obesity: increases the risk of Charcot by 1.5 times compared to non-obese individuals [47].
- Previous Trauma: traumatic events are very frequently reported as triggers for acute Charcot [48].

The most affected site is the midfoot (up to 86% of cases), followed by the forefoot and ankle; involvement is usually unilateral, but bilateral forms occur in approximately 9–20% of patients [46–49].

Pathophysiology.

Acute Charcot neuroarthropathy is a complex process triggered by neuropathy, microtrauma, and an uncontrolled inflammatory response [50]. A triggering event (even an underestimated trauma) initiates a disproportionate local inflammatory cascade, releasing cytokines that disrupt the balance between RANKL (which stimulates osteoclasts) and osteoprotegerin [51]. This results in a pathological activation of osteoclasts, which begin to aggressively resorb bone, weakening the skeletal structure of the foot. This leads to microfractures under normal loads and potential bone collapse if the patient continues to ambulate [52, 53].

Clinical presentation and diagnosis.

- Acute Charcot foot presents with peripheral inflammation characterized by:
- Edema.
- Erythema (Redness).
- Hyperthermia (Heat).
- Potential foot deformities located in the forefoot, midfoot, and rearfoot, alone or together.
- Additional elements: pain and fever.

**Fig. 4:**
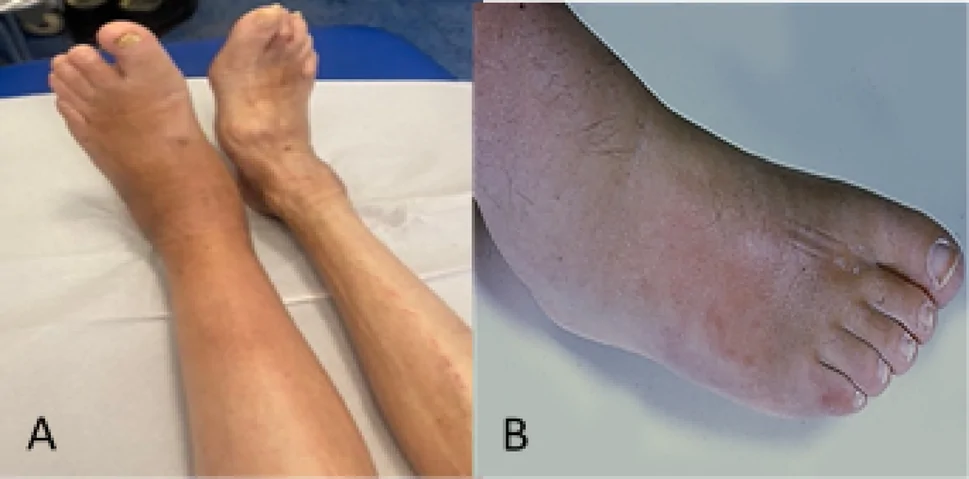
**A**: left foot acute Charcot with oedema of whole leg. **B**: right foot acute Charcot with oedema and midfoot redness

Diagnostic difficulty arises because it perfectly mimics more common conditions such as infectious cellulitis, gout, traumatic sprain, thrombophlebitis, and deep vein thrombosis (DVT), often leading to diagnostic delays that irreparably compromise the structural integrity of the foot (Fig. 4).

Therefore, when a clinician encounters a patient with neuropathy presenting an edematous, erythematous, and hyperthermic foot, an immediate clinical alarm must be triggered. Charcot neuroarthropathy must be the first hypothesis to be excluded. Time is fundamental at this stage to prevent significant bone deformities.

Diagnosis in the acute phase is clinical:

- Careful Anamnesis: onset and evolution of the clinical picture.
- Thermal Gradient: an objective and easily detectable indicator. A skin temperature difference of > 2°Ccompared to the contralateral foot, measured with an infrared thermometer at corresponding points, is highly suggestive of an active Charcot pattern [54].
- Pain: normally absent in neuropathic patients, the perception of pain is a major warning sign.
- Radiological Signs: plain X-rays may be negative in early stages. In that case, an MRI of the foot is the referral diagnostic procedure. Early signs include soft tissue edema and bone marrow edema. Other late signs might be focal osteopenia (localized demineralization due to hyperemia), subtle subluxations, widening of metatarsal spaces, subchondral fragmentation, and cortical disruption [55].

Management.

During the stage of “clinical suspicion,” it is essential not to delay conservative treatment. The most appropriate treatment involves non-weight-bearing to limit or halt the osteodestructive process [56].

Waiting for classic radiographic signs of fragmentation or midfoot collapse (the “rocker-bottom” foot) means intervening when the damage is already consolidated, and the risk of ulceration and subsequent amputation is extremely high.

**Fig. 5:**
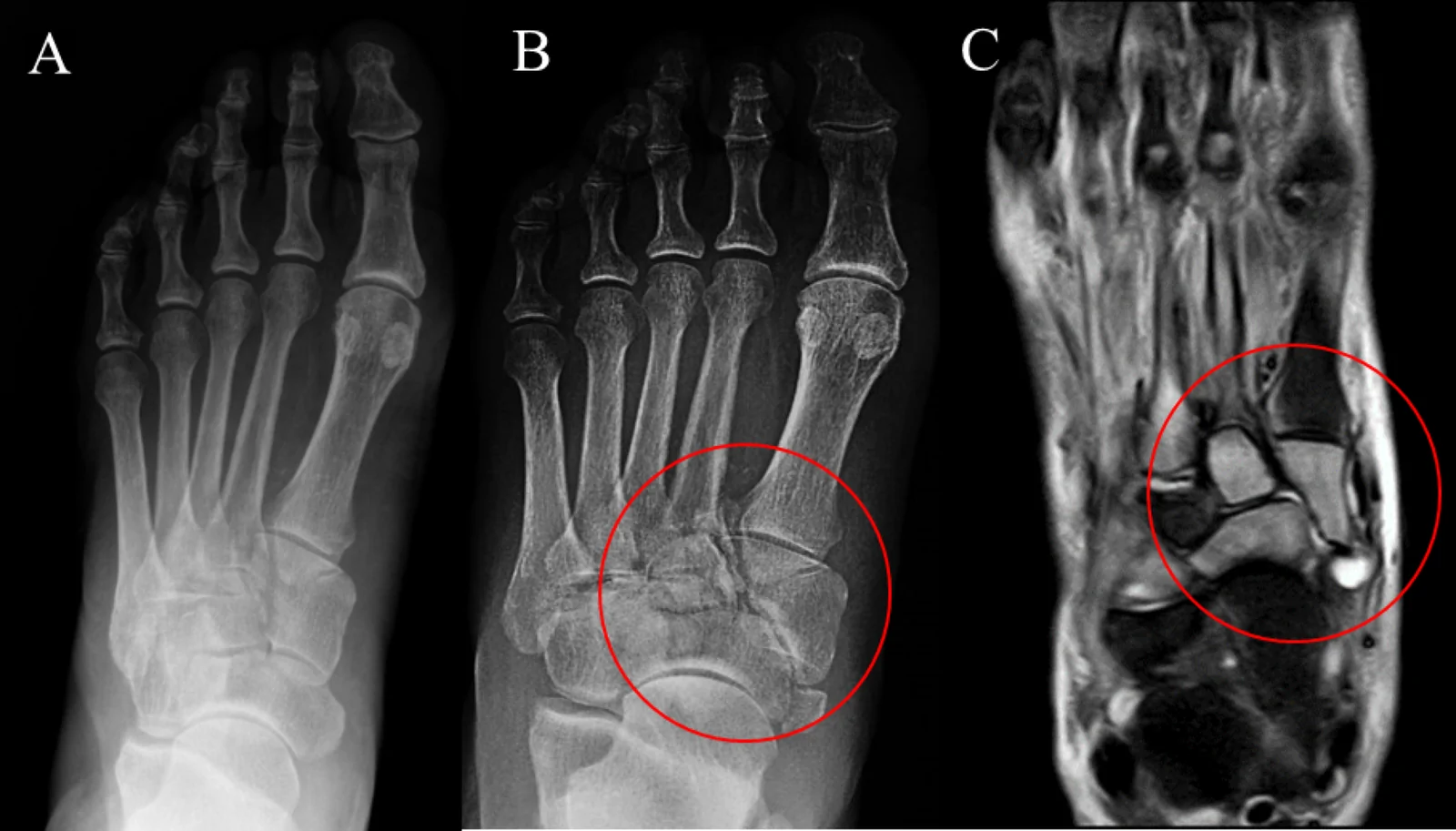
**A**: x-ray at symptoms beginning, **B**: x-ray after 2 months without offloading, **C**: MRI with evidence of bone oedema localized at midfoot

Actions in case of clinical suspicion:

- immediate prescription of complete non-weight-bearing of the affected limb. Advising the patient to simply “walk less” is insufficient; weight-bearing must be strictly avoided until specialist evaluation.
- Urgent referral (preferably within 24–48 h) to a multidisciplinary second- or third-level diabetic foot center. These facilities can initiate immobilization protocols using Total Contact Casts (TCC) or dedicated braces and perform advanced imaging studies such as MRI, which can detect bone marrow edema before radiographic abnormalities become evident on plain X-rays (Fig. 5).

Early recognition of acute Charcot foot means transforming a potential permanent disability into a manageable condition, preserving the patient’s quality of life and mobility (Table 3).

**Table 3:** Acute charcot foot: clinical features, diagnosis and management

Clinical Presentation • Edema • Erythema (Redness) • Hyperthermia (Heat) • Deformities • Additional element: pain	Diagnosis • Anamnesis: Onset and evolution of the clinical picture. • Thermal Gradient: skin temperature difference of >2° compared to the contralateral foot • Pain: abnormally presence in neuropathic • Radiological Signs: early signs include soft tissue edema and late signs include focal osteopenia, subtle subluxations, widening of metatarsal spaces, subchondral fragmentation, and cortical disruption	Management in the case of clinical suspicion: • Prescription of immediate and total non-weight-bearing of the affected limb. • Urgent referral to a multidisciplinary second- or third-level diabetic foot center.

### Severity and time-driven referral pathway

**Fig. 6:**
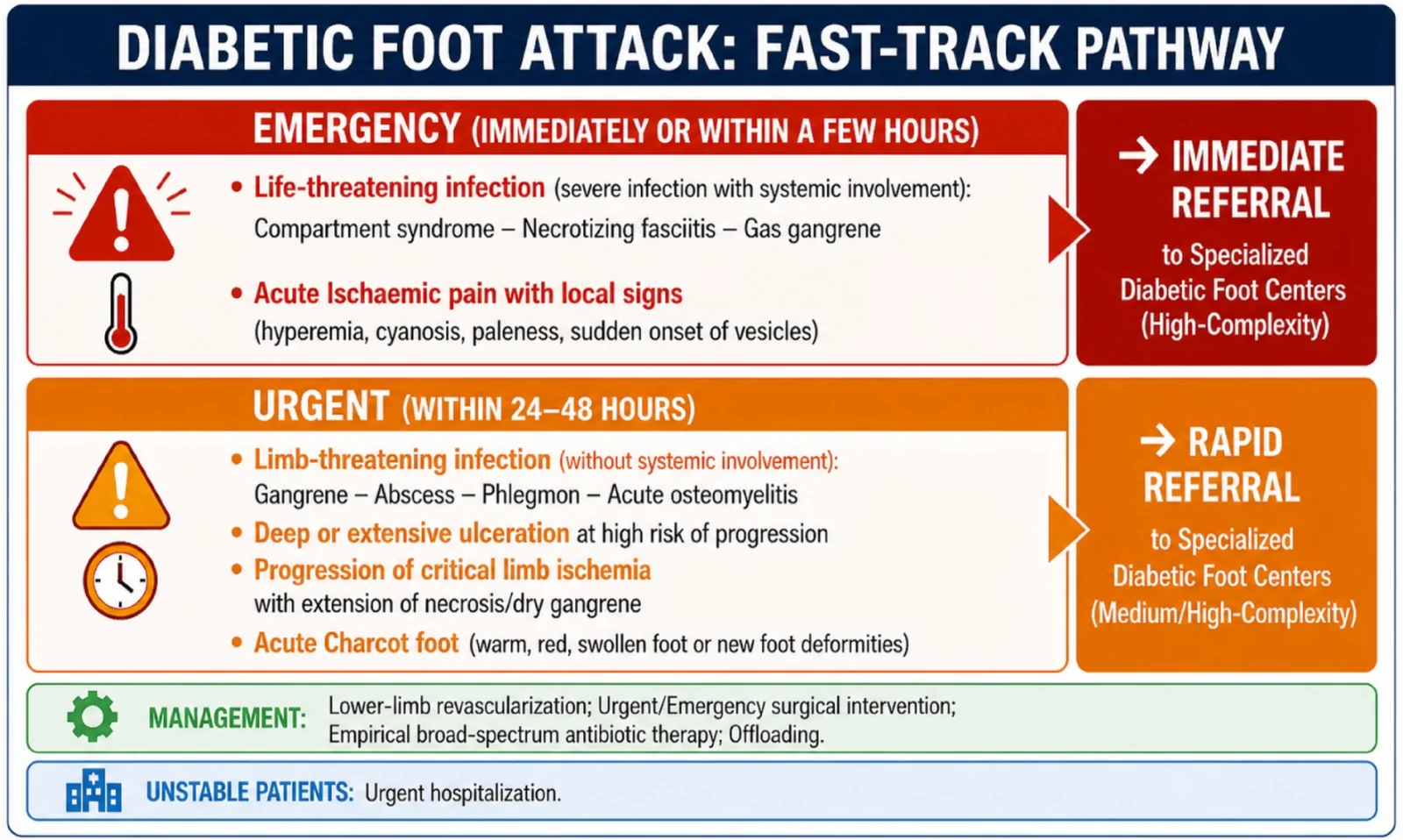
Clinical fast-track referral model for diabetic foot attack

This fast-track model defines referral pathways to specialized diabetic foot care centers according to clinical presentation, with the primary aim of ensuring rapid recognition and timely referral of patients at risk of limb loss or life-threatening complications. Referral decisions are based on the degree of urgency or emergency related to the severity of each clinical condition, thereby optimizing access to the appropriate level of care within the healthcare network, according to the organization and expertise of individual centers (Fig. 6).

## Discussion

The major clinical presentations of DFA, including ischaemia, infection, and acute Charcot neuroarthropathy, should all be considered time-dependent and potentially limb-threatening conditions [3, 21]. In this context, ulceration frequently represents the clinical entry point through which these conditions develop and progress.

Within the proposed fast-track model for DFA, foot ulceration has been deliberately included as a key clinical entry point because of its central role as both a trigger and amplifier of the pathological cascade. An apparently uncomplicated ulcer may represent the initial driver of downstream complications, including infection and ischaemia, and may also coexist with or complicate the evolving phases of acute Charcot neuroarthropathy.

Accordingly, timely and appropriate management of foot ulceration is essential to prevent or limit progression of the DFA cascade. Delays in recognition or treatment may facilitate progression toward limb-threatening conditions, whereas early stratification and targeted intervention are critical determinants of limb salvage.

DFU characteristics, including size, depth, infection, vascular impairment, and anatomical location (such as heel involvement), are major determinants of prognosis and require early specialist evaluation to guide appropriate management [57].

Ischemia is a major determinant of poor outcomes, including non-healing, limb loss, and mortality [23]. Both acute limb ischaemia and progression of chronic limb-threatening ischemia are characterized by rapid tissue deterioration and require urgent vascular assessment and timely revascularization to preserve the limb [58, 59].

Infection is a frequent and critical cause of clinical deterioration, often superimposed on pre-existing ulcers and capable of extending to deep tissues, including bone. The clinical spectrum ranges from superficial infection to severe, potentially life- and limb-threatening conditions such as abscess, phlegmon, necrotizing fasciitis, and gangrene. Given its strong association with rapid progression, infection requires early recognition, prompt antimicrobial therapy, and timely surgical evaluation, with escalation to urgent intervention in the presence of severe features [38]. Delays in diagnosis and treatment remain major modifiable factors associated with adverse outcomes [30].

Acute Charcot neuroarthropathy, although less common, represents a high-risk and frequently underdiagnosed condition. Its early presentation can mimic infection or other inflammatory disorders, leading to diagnostic delay. Immediate clinical suspicion, early offloading, and urgent referral are essential to prevent irreversible structural collapse of the foot [60].

All components of DFA—ulceration, ischaemia, infection, and acute Charcot neuroarthropathy—must be systematically characterized using standardized diagnostic criteria and validated classification systems, which support risk stratification and drive clinical decision-making by using the fast-track pathway.

Each of these conditions may rapidly progress; therefore, early identification of clinical warning signs is crucial to assess severity and to distinguish patients requiring prompt referral to a dedicated specialized DFS. This represents a key step in the management pathway, enabling access to vascular assessment, surgical expertise, advanced wound care, offloading strategies, and coordinated multidisciplinary care.

## Conclusions

Delays in recognition and referral continue to represent critical determinants of poor outcomes. In this context, the proposed fast-track pathways should not be viewed simply as organizational tools.

The Fast-track approaches redefine priorities, placing timeliness at the center of care for acute diabetic foot conditions associated with a high risk of limb loss in the absence of prompt and appropriate management. Further surveys in primary care and other healthcare settings will be useful to assess the appropriateness of the proposed model, its feasibility, and potential improvements in the management of acute diabetic foot.

## Supplementary Information

Below is the link to the electronic supplementary material.

Supplementary Material 1
