# Supplementary material for: The acute diabetic foot: different clinical patterns with a dedicated fast-track pathway
Source: Acta Diabetol. 2026 Jul 14;63(11):1875–86. doi: 10.1007/s00592-026-02746-w (PMC13690879; doi:10.1007/s00592-026-02746-w)
Supplement: Supplementary file 1 [file 592_2026_2746_MOESM1_ESM.pdf]

The authors have no conflicts of interest to declare that are relevant to the content of this article.
